# Influence of Simplified Microbial Community Biofilms on Bacterial Retention in Porous Media under Conditions of Stormwater Biofiltration

**DOI:** 10.1128/Spectrum.01105-21

**Published:** 2021-10-27

**Authors:** Yue Zhang, Yan He, Eric G. Sakowski, Sarah P. Preheim

**Affiliations:** a Department of Environmental Health and Engineering, Johns Hopkins University, Baltimore, Maryland, USA; University of Minnesota

**Keywords:** *E. coli*, porous media, stormwater biofilters, biofilm, microbial community, microbial interaction, community structure

## Abstract

Porous media filters are used widely to remove bacteria from contaminated water, such as stormwater runoff. Biofilms that colonize filter media during normal function can significantly alter performance, but it is not clear how characteristics of individual populations colonizing porous media combine to affect bacterial retention. We assess how four bacterial strains isolated from stormwater and a laboratory strain, Pseudomonas aeruginosa PAO1, alter Escherichia coli retention in experimental sand columns under conditions of stormwater filtration relative to a clean-bed control. Our results demonstrate that these strains differentially affect E. coli retention, as was previously shown for a model colloid. To determine whether E. coli retention could be influenced by changes in relative abundance of strains within a microbial community, we selected two pairs of biofilm strains with the largest observed differences in E. coli retention and tested how changes in relative abundance of strain pairs in the biofilm affected E. coli retention. The results demonstrate that E. coli retention efficiency is influenced by the retention characteristics of the strains within biofilm microbial community, but individual strain characteristics influence retention in a manner that cannot be determined from changes in their relative abundance alone. This study demonstrates that changes in the relative abundance of specific members of a biofilm community can significantly alter filter performance, but these changes are not a simple function of strain-specific retention and the relative abundance. Our results suggest that the microbial community composition of biofilms should be considered when evaluating factors that influence filter performance.

**IMPORTANCE** The retention efficiency of bacterial contaminants in biofilm-colonized biofilters is highly variable. Despite the increasing number of studies on the impact of biofilms in filters on bacterial retention, how individual bacterial strains within a biofilm community combine to influence bacterial retention is unknown. Here, we studied the retention of an E. coli K-12 strain, as a model bacterium, in columns colonized by four bacterial strains isolated from stormwater and P. aeruginosa, a model biofilm-forming strain. Simplified two-strain biofilm communities composed of combinations of the strains were used to determine how relative abundance of biofilm strains affects filter performance. Our results provide insight into how biofilm microbial composition influences bacterial retention in filters and whether it is possible to predict bacterial retention efficiency in biofilm-colonized filters from the relative abundance of individual members and the retention characteristics of cultured isolates.

## INTRODUCTION

Bacterial contaminants pollute water bodies (1), prevent water reuse (2, 3), and severely threaten public health (4). Pathogen contamination, including bacterial pathogens, is one of the most frequent causes of water impairments. The U.S. Environmental Protection Agency, in their Water Quality Assessment and TMDL Information from 2017 (https://ofmpub.epa.gov/waters10/attains_index.home), reported that about 17% (187,872 miles) of assessed U.S. rivers and streams were contaminated by pathogens. It is estimated that diseases transmitted by waterborne pathogens contribute to approximately 500,000 hospital visits annually and cost nearly 4 billion U.S. dollars every year (4).

In urban areas, stormwater runoff is often polluted by bacterial contaminants and considered to be the major source of contamination of surrounding water bodies (2, 5–7). During storm events, stormwater runoff mobilizes various contaminants while flushing impervious surfaces. To mitigate the risk of contamination from stormwater, a variety of techniques to clean stormwater using porous media (2, 8–10) have been applied. Biofilters (also known as bioretention, biofiltration, rain gardens, etc.) are one of the most commonly used techniques in urbanized areas to remove bacteria and other types of contaminants (e.g., viral and other particulate contaminants, oil and grease, nitrogen species, heavy metals) (7, 11–14). The main feature of typical biofilters is an underground cell filled with sands or other porous media, where contaminants are designed to be captured and removed from stormwater runoff. However, the removal efficiency of bacterial contaminants in deployed filters can be variable and unpredictable (10, 15). Such variability is often attributed to a series of factors, including specifications of filter design (e.g., the dimensions of filter, the component of porous media) (6, 16, 17), influent water chemistry (e.g., pH, salinity, the presence of natural organic matters) (18–20), environmental conditions (e.g., interval between dry and wet event, temperature) (21, 22), colonization of the media surface by microbial communities that disperse into filters with influent water during operation (23–29), and the degradation or predation of captured bacterial contaminants within biofilm (e.g., protozoa grazing and viral infection of pathogens) (30).

Previous studies have demonstrated that biofilm development in porous media alters colloid and microbial retention, but the direction and magnitude likely depend on a combination of particulate characteristics, biofilm composition, and other physical and chemical factors (23–27, 31, 32). For instance, a study simulating groundwater filtration in the subsurface revealed that biofilms formed by groundwater microbiota improved Escherichia coli retention rate (i.e., the percentage of *E. coli* cells retained on filter) by 38% relative to a control (31). Another study found that biofilms formed from unchlorinated tap water from a river increased the retention efficiency of Cryptosporidium parvum by 26% in sand filters and by 31% in granular activated carbon filters relative to a control while simulating drinking water filtration (32). While these studies found that biofilm development enhanced microbial retention in filters, other studies indicated that microbial retention might be significantly hindered by biofilms (23, 25). For example, the development of Pseudomonas aeruginosa biofilm in columns filled with glass beads was found to decrease the retention efficiency of Cryptosporidium parvum oocysts by 28% relative to a control (25). The variability induced by biofilm formation will make prediction and control of retention of microbial contaminants in deployed filters difficult without a fundamental understanding of factors that create this variability.

The variation in bacterial retention induced by biofilms might be attributable to the high variability in strain-specific characteristics of biofilm-forming bacteria, but the extent to which environmental bacteria can influence bacterial retention is unknown. Our previous study investigating the impact of biofilm formation by four bacterial strains isolated from stormwater on the retention of a model colloid (carboxyl-modified-latex beads) in porous media showed that interstrain variation in colloid retention efficiency was as high as 80% (33). A substantial amount of variability in colloid retention in that study could be explained by strain-specific differences in adhesive forces between the biofilm and particle, measured by atomic force microscopy, which was assumed to be related to strain-specific surface characteristics such as the length and composition of biopolymers on biofilm surface (33). However, it is not clear if the findings are applicable to bacterial retention because of the differences in surface characteristics (33–35), shapes, and sizes between the model colloid and bacterial cells (36). Previous studies (37, 38) have also demonstrated that biofilms formed by model laboratory strains, such as Pseudomonas aeruginosa PAO1 and PDO300, have different bacterial retention efficiencies, attributable to their variation in surface characteristics (e.g., biofilm surface charge, hydrophobicity, and biopolymer properties). For example, the ionization of macromolecules on biofilm surfaces induces electric-double-layer force that attracts or repulses particulates during filtration (39, 40). Hydrophobic biofilm surfaces remove water molecules from the surface and hence facilitate the adhesion to apolar surface sites of particulates (41, 42). Additionally, long biopolymers protruding from biofilm surface might also extend beyond the range of electric-double-layer force and hence interact with particulates by themselves (33, 37, 40). However, because laboratory strains usually have a prolonged history of laboratory cultivation or genetically modified traits, it is not clear if the findings with laboratory strains can be applied broadly to environmental biofilms (43–45). Therefore, more studies are needed to evaluate the impact of biofilms formed by environmentally relevant strains on bacterial retention.

Biofilms in the environment are composed of complex microbial communities, but it is not clear how biofilm microbial community composition influences bacterial retention in filters. By testing the retention of various types of particles in single-strain biofilm-colonized filters, previous studies (27, 33, 38, 46, 47) have demonstrated that biofilms influence the retention of particles by covering and modifying porous media surfaces with their own specific surface characteristics. If the relative abundance of a population in a biofilm microbial community relates to the proportion of porous media surface area covered by that population, the impact of biofilms of complex microbial communities on bacterial retention will be closely related to the physicochemical characteristics of all constituent populations and their relative abundance. In other words, higher relative abundance of a population that is strongly adhesive to bacterial contaminants could lead to higher bacterial retention efficiency in the filter. On the other hand, if the surface area covered by a population does not scale with its relative abundance, its impact on bacterial retention will be disproportional or unrelated to its relative abundance. Therefore, understanding how biofilm microbial composition influences bacterial retention will provide insights into the prediction of bacterial retention in deployed filters.

The present study aims to evaluate how biofilm-forming environmental bacterial isolates influence bacterial retention and to understand whether changes in relative abundance of members within simplified microbial communities alter bacterial retention. This study was performed under the conditions of simulated stormwater biofiltration because stormwater biofilters are frequently used to treat bacterial contaminants in stormwater runoff. First, we assessed the impact of biofilm formation in porous media on the retention of a bacterial contaminant, E. coli K-12 MG1655, with four biofilm-forming strains isolated from stormwater (SW1 to 4) and a model biofilm-forming isolate, P. aeruginosa PAO1. The five selected biofilm-forming strains, which were shown to have different retention efficiencies for a model colloid in our previous study (33), also created strain-specific retention rates of E. coli in this study. To elucidate how biofilm community composition influences bacterial retention, we measured E. coli retention in a series of columns colonized by simplified communities composed of two strains at different relative abundances. The results demonstrate that E. coli retention in porous media can be significantly influenced by the microbial composition of the simplified biofilm communities but cannot be directly predictable from the relative abundance and retention characteristics of each strain.

## RESULTS

### E. coli retention in sand columns colonized by single isolates.

**FIG 1:**
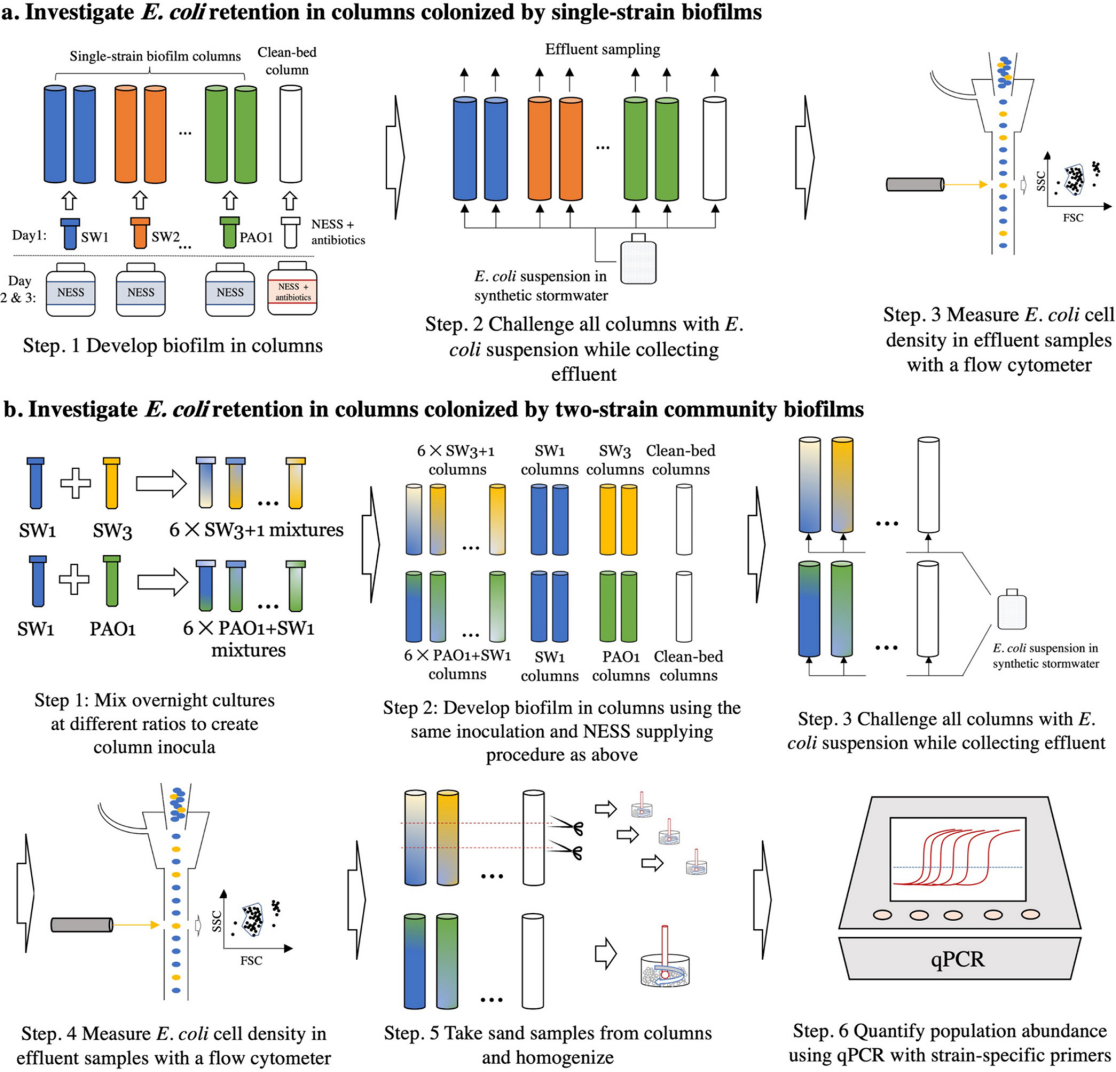
A flowchart of the major steps in experimental methodology.

To investigate the extent to which stormwater isolates influence bacterial retention in porous media, we tested the retention of an E. coli K-12 strain in laboratory-scale sand columns colonized by four strains isolated from sand columns inoculated with stormwater runoff (SW1 to 4) and a model laboratory strain, P. aeruginosa PAO1 (abbreviated as PAO1 hereafter), individually (Fig. 1a). Nutrient-enriched synthetic stormwater (NESS) (33) was used as growth medium for expediting biofilm development while holding constant the most salient aspects of stormwater chemistry (e.g., pH, ionic strength). A clean-bed (no biofilm) column was included in each batch of experiments, where no isolate was inoculated and kanamycin sulfate antibiotic was added to the filtrate during the biofilm growth period to maintain sterile conditions.

**FIG 2:**
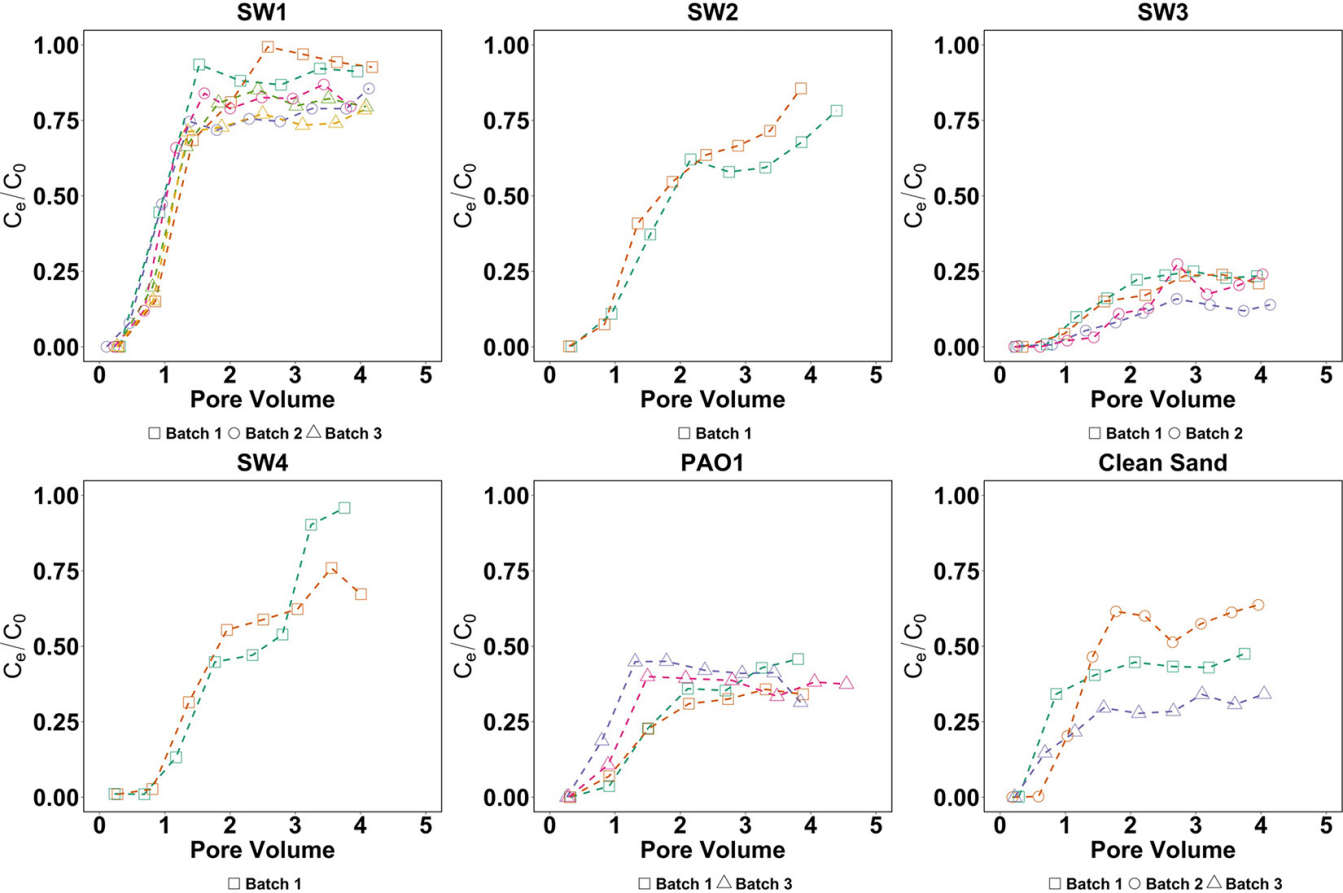
The breakthrough curves of E. coli in sand columns colonized by stormwater strains (SW1 to 4), P. aeruginosa PAO1, and clean-bed sand columns. *x* axis shows the volume of E. coli suspension injected into each column in the unit of the pore volume; *y* axis shows the normalized E. coli concentration in the effluent (E. coli concentration in effluent divided by the one in influent, C_e_/C_0_). Breakthrough curves with different colors in every subplot represent E. coli transport in different columns.

Biofilm development of environmental isolates in porous media alters the transport of E. coli cells compared to that of clean-bed controls (Fig. 2). The normalized E. coli concentration in the effluent (i.e., E. coli concentration in effluent divided by that in influent [C_e_/C_0_]) is plotted against the volume of E. coli suspension injected into each column in the unit of pore volume (PV). Variability in the retention of E. coli on clean-bed columns was larger between experimental batches (Fig. 2, clean sand) than within the same batch (Fig. S1). Larger variability between batches than within batches might be attributable to a certain level of inconsistency in porous media preparation. Nevertheless, this variation was reduced after biofilm development (SW1 to 4 and PAO1 in Fig. 2), likely because biofilm development covered and modified the sand surfaces. To account for the variability across the three batches, we pooled all batches for further analysis.

We observed differences in E. coli transport across columns colonized by individual strains and high reproducibility between replicate columns colonized by the same strains (Fig. 2). Except the columns modified by SW2 and SW4, all other columns had normalized E. coli effluent concentration that reached a plateau and remained relatively stable after 2 PV of E. coli injection. SW2 and SW4 columns showed two separate breakthrough events within 4 PV of E. coli injection. The second breakthrough event occurred at approximately 1 PV after the first breakthrough event, and the normalized effluent concentration was likely rising toward 100%, although it was not captured within 4 PV E. coli injection. The SW3 and PAO1 columns had the lowest normalized effluent concentration among all strains.

The normalized effluent concentration after 2 PV was averaged to calculate an E. coli retention efficiency to represent strain-specific E. coli retention characteristics (Fig. S2). E. coli retention efficiency was significantly different between strains (*P* < 0.05, one-way analysis of variance [ANOVA]). E. coli retention efficiency of clean-bed columns was 0.56 ± 0.14 after accounting for the variability across the three experiments. Even with the high variability in clean-bed columns, SW1 columns were still significantly different from the clean-bed columns (*P* < 0.05, Welch *t* test). SW1 columns had the lowest mean E. coli retention efficiency (0.17 ± 0.07), significantly different from that of all other strains (*P* ≤ 0.05). The E. coli retention efficiencies in SW2 (0.32 ± 0.05) and SW4 columns (0.31 ± 0.04) were very similar, and both were significantly different from PAO1 and SW3 (*P* < 0.05). The E. coli retention efficiency of PAO1 (0.63 ± 0.03) was significantly different from that of SW3 (*P* < 0.05), which had the highest E. coli retention efficiency (0.80 ± 0.04) among all isolates. SW2 and SW3 are closely related (under the same genus of *Chryseobacterium* [33]) but had significantly different E. coli retention efficiencies (difference between strains of 0.48 ± 0.09, *P* < 0.05). Similarly, PAO1 and SW4 are also closely related and belong to the genus Pseudomonas, while they had significant differences in E. coli retention efficiency (difference between strains of 0.32 ± 0.07, *P* < 0.05). Overall, the results demonstrate that biofilm development by environmental isolates can alter E. coli retention, but the retention characteristics of natural isolates are not strongly phylogenetically constrained.

In order to determine if the retention characteristics of biofilm-forming isolates are consistent for different particulates, we compared the retention efficiency of E. coli and that of carboxyl-modified-latex (CML) beads (Fig. S2), of which the data were acquired from our earlier study (33). The retention of CML beads was measured under experimental settings similar to those in the current study (e.g., porous media, column dimensions, flowrate of filtrate) but different in filtrate chemistry (i.e., the filtrate suspending CML beads contains 3 g/liter of yeast extract whereas the filtrate suspending E. coli does not). The retention efficiencies between the two particulates were significantly different in clean-bed (*P* < 0.05), SW2 (*P* < 0.05), SW4 (*P* < 0.05), and PAO1 columns (*P* < 0.001) but similar in SW1 and SW3 columns (*P* > 0.05). The results indicate that relative retention efficiencies across the five strains were different for E. coli and CML beads, although some strains had similar retention efficiencies between the two particulates.

### E. coli retention in sand columns colonized by two-strain communities of various strain abundance ratios.

To investigate how changes in relative abundance of populations within biofilm microbial communities influence bacterial retention, we prepared a series of columns with simplified communities containing two strains at different relative abundances (Fig. 1b). Two different two-strain communities were assessed, one with a combination of SW3 and SW1 (abbreviated as SW3+1) and another with a combination of PAO1 and SW1 (abbreviated as PAO1+SW1). These strains were chosen because they had the largest differences in E. coli retention characteristics and stable breakthrough curves. To manipulate the relative abundance of each strain in biofilm microbial communities on the columns, we changed the amount of culture of each strain added to inoculate the column. To quantify the abundance of each strain in columns after 3 days of growth, we designed strain-specific primers (Table S1) targeting a region of the 16S rRNA gene unique to each strain, which had high specificity to the targeted bacteria (Table S2).

**FIG 3:**
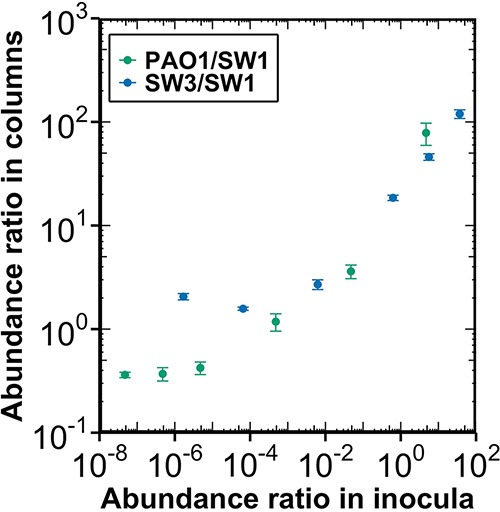
The variation of biofilm composition with changing inoculum abundance ratios in simplified bacterial communities. The abundance on both *x* and *y* axes was measured with quantitative PCR using strain-specific primers. *x* axis represents the abundance ratios in each column’s inoculum, which were calculated using the measured abundance of PAO1, SW1, and SW3 in their overnight cultures before mixing and the corresponding mixing ratios; *y* axis represents the observed abundance ratios of biofilm communities on columns after the incubation period. Horizontal error bars, which represent the standard deviation of three technical replicate measurements in quantitative PCR, are not presented because they are smaller than the symbols. The vertical error bars represent the standard deviation of technical replicate measurements in quantitative PCR.

Microbial composition of biofilm community colonizing the sand columns was manipulated by varying the inoculum microbial composition, although not with a direct correspondence (Fig. 3 and Fig. S3 and S4). The observed biofilm microbial composition in columns is a result of changes to the inoculum composition and the strains’ relative ability to grow in our nutrient-supplemented media, since nutrient load can influence the microbial composition of biofilm community by promoting the growth or detachment of different species in biofilms (48–51).

**FIG 4:**
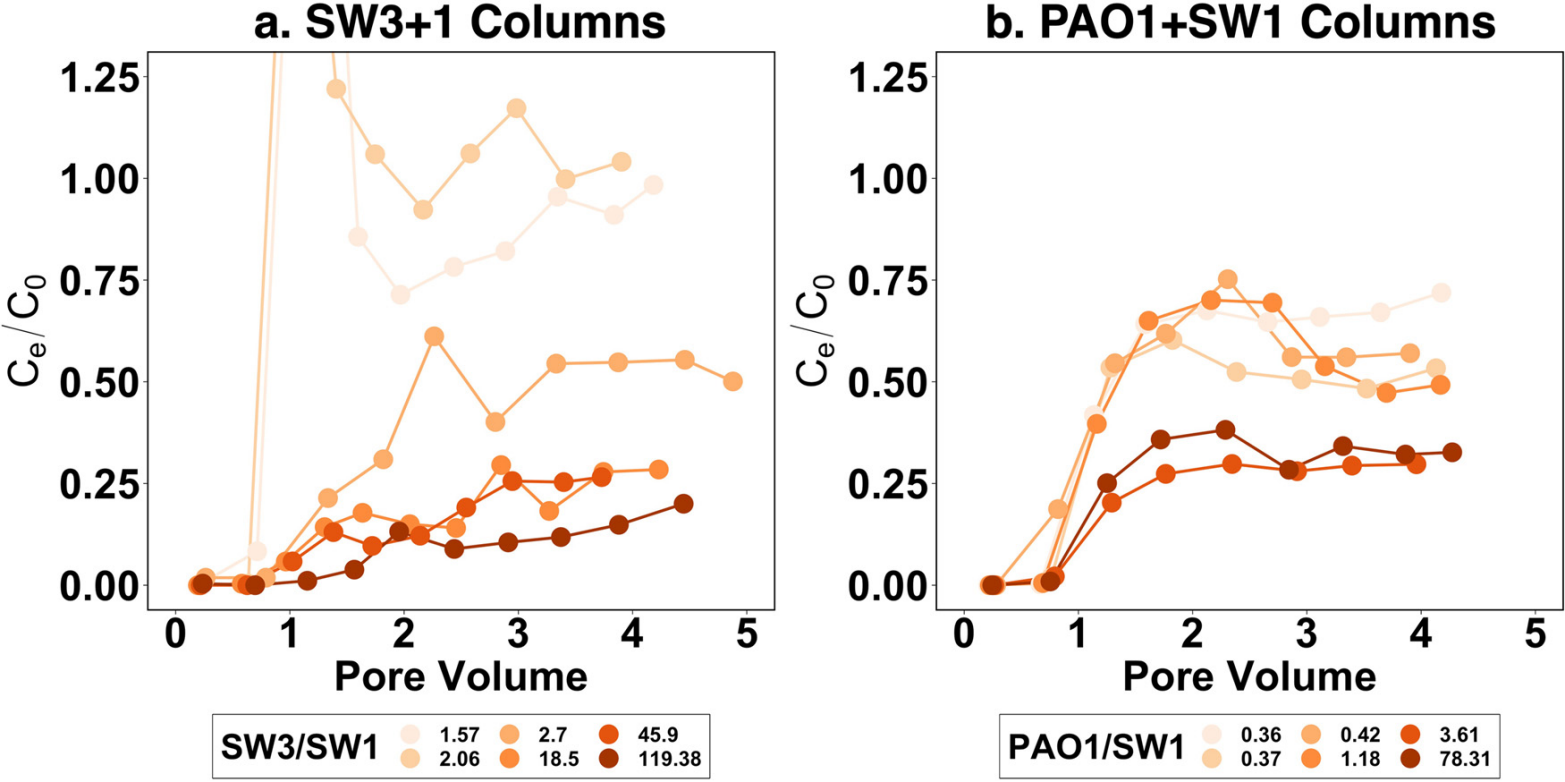
The breakthrough curves of E. coli in columns colonized by SW3+1 communities (a) and those colonized by PAO1+SW1 communities (b), color-coded by relative abundance ratios of constituent populations in each column. *x* axis shows the volume of E. coli suspension injected into each column in the unit of the pore volume; *y* axis shows the normalized E. coli concentration in the effluent (E. coli concentration in effluent divided by that in influent, C_e_/C_0_). Strain ratios for two-strain communities were calculated by dividing the concentration of amplicons from one strain by the other.

To investigate how the microbial composition of biofilm communities influences E. coli retention, E. coli was added to the sand columns colonized by the two-strain communities with various strains’ relative abundances. Breakthrough curves show a transition between higher transport (less E. coli retention on the column), characteristic of SW1, to lower transport (more E. coli retention on the column), characteristic of SW3 and PAO1, as the relative abundance of SW1 decreased across columns (Fig. 4). Although normalized E. coli effluent concentration fluctuated beyond 100% at the beginning of the E. coli injection in a SW3+1 column (Fig. 4a, breakthrough curve labeled with SW3/SW1 equal to 1.57), it was relatively stable after 2 PV of E. coli injection. For the purpose of comparing across columns, we took the average of the normalized E. coli effluent concentration after 2 PV to calculate the E. coli retention efficiency for each column, which is consistent with the analysis of single-strain colonized columns.

**FIG 5:**
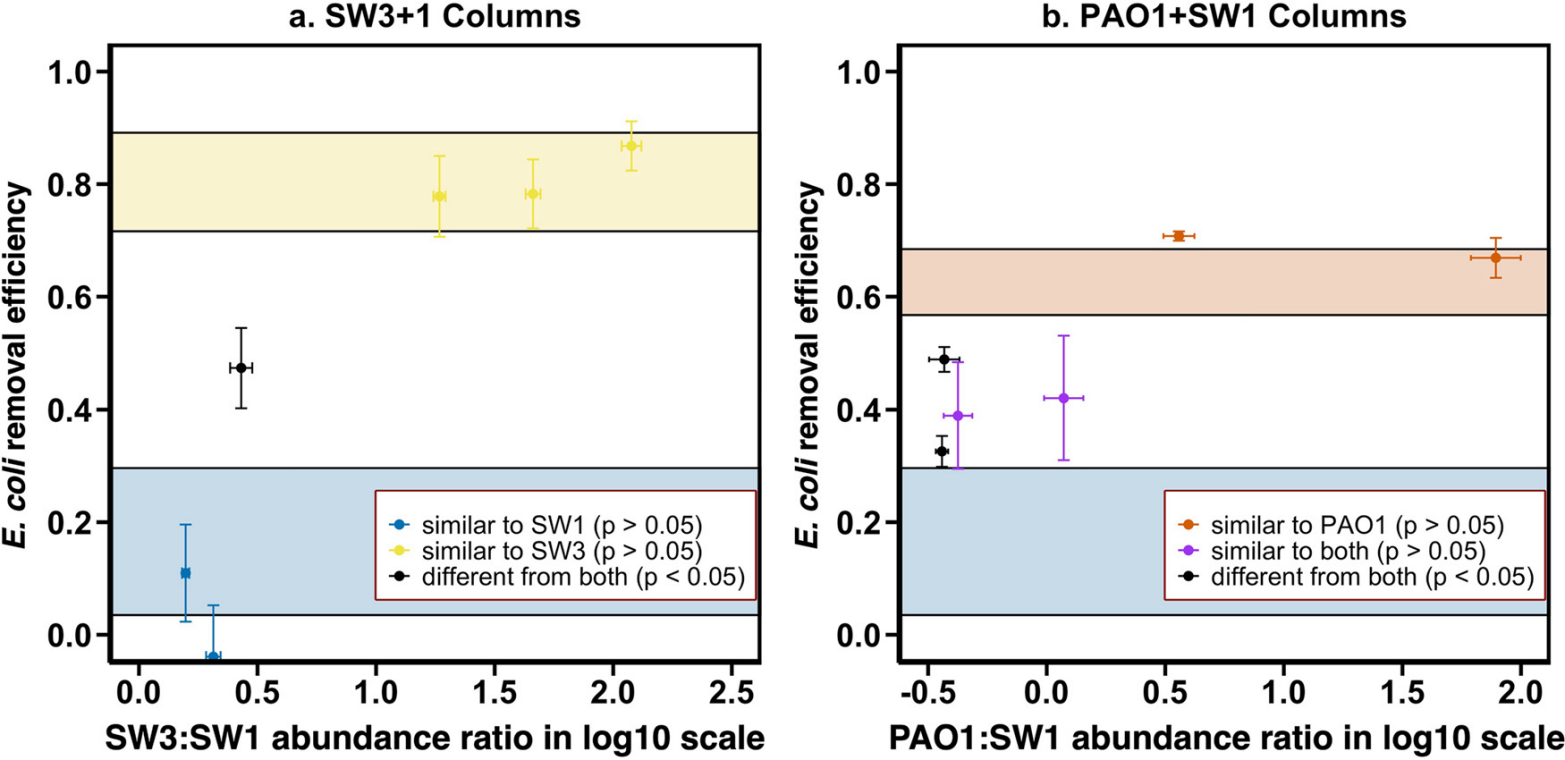
The variation of E. coli retention efficiency of columns colonized by SW3+1 and PAO1+SW1 communities at different abundance ratios between constituent populations in column. Six columns were colonized by SW3+1 community (a), and the other six columns were colonized by PAO1+SW1 community (b). Vertical error bars represent the standard deviation of E. coli retention efficiency in a column after 2 PV of E. coli injection. On the *x* axis, the abundance was measured with quantitative PCR using strain-specific primers. Horizontal error bars represent the standard deviation of three technical replicate measurements in quantitative PCR. The yellow, blue, and red boxes represent the E. coli retention efficiency (mean ± 2 standard deviation) of columns colonized by SW3, SW1, and PAO1, respectively.

Biofilm formation with two-strain communities (i.e., SW3+1 and PAO1+SW1) induced significant differences in E. coli retention across columns, more than the differences observed for biofilms developed by a single strain (Fig. S5). As the conditions were identical across all the columns and no biological contamination (e.g., the dispersal of bacteria from ambient environment into columns) was detected, the difference in E. coli retention efficiency between columns can be attributed to the microbial composition of colonizing biofilm communities. To elucidate how E. coli retention changes as a function of colonizing biofilm microbial composition, we plotted E. coli retention efficiency against the abundance ratio between the two constituent community members for each column (Fig. 5). If retention of E. coli changed predictably as a function of ratio of relative abundance of strains in the community, the curves for each pair of strains would be similar. However, there were substantial differences in the shapes of these curves. For columns colonized by SW3+1 communities, when the SW3/SW1 abundance ratio was low (2.06 or less, blue cross marks in Fig. 5a), the E. coli retention efficiency of these columns was not significantly different from that of SW1-only columns (*P* > 0.05, Welch *t* test). When the SW3/SW1 abundance ratio was high (18.5 or more, yellow cross marks in Fig. 5a), the E. coli retention efficiency of these columns was not significantly different from that of SW3-only columns (*P* > 0.05). Only one column had an E. coli retention efficiency significantly different from that of both SW1-only and SW3-only columns (SW3/SW1 ratio equal to 2.7, *P* < 0.05, black cross mark in Fig. 5a). For columns colonized by PAO1+SW1 communities, 4 columns had E. coli retention efficiency in between PAO1-only and SW1-only columns, although only two columns (black cross marks in Fig. 5b) were significantly different from both PAO1-only and SW1-only columns (*P* < 0.05). The other two columns (purple cross marks in Fig. 5b) were not statistically different from either PAO1-only or SW1-only columns (*P* > 0.05), likely due to the fluctuations in the breakthrough curves, indicated by the large error bars. The PAO1:SW1 abundance ratios in these four columns ranged from 0.36 to 1.18.

### The spatial distribution of strains within SW3+1 community columns.

**FIG 6:**
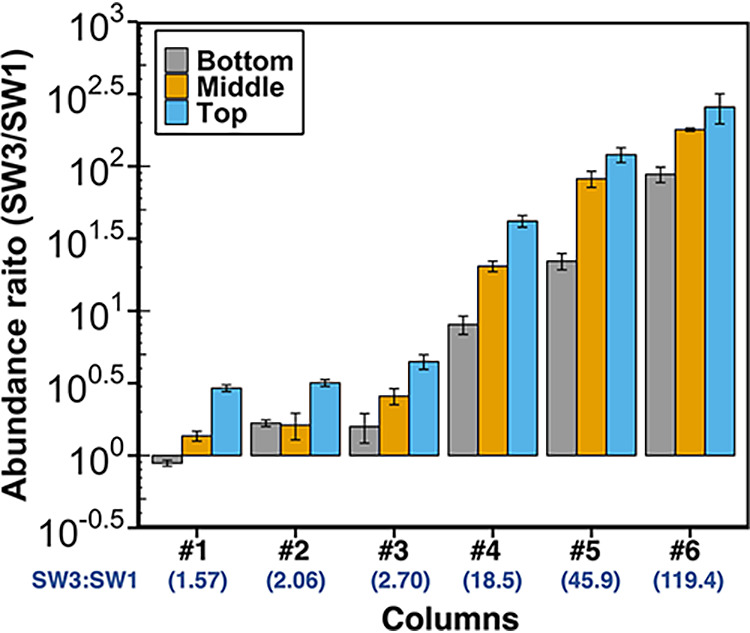
The distribution of strains SW3 and SW1 in the top, middle, and bottom sections of the column demonstrates nonhomogeneous distributions of strains in mixed community columns. *x* axis shows a total of six columns colonized by the SW3+SW1 community (numbers 1 to 6) which vary according to the ratio of strains in the inoculum of each column. Numbers in parentheses indicate the SW3/SW1 abundance ratio of the column measured by quantitative PCR (qPCR) using strain-specific primers. *y* axis shows the observed abundance ratios of SW3 and SW1 across sections of the column. Error bars represent the standard deviation of three technical replicate measurements in quantitative PCR.

To explore whether the spatial distribution of strains in sand columns is influenced by the presence of other strains, we analyzed the community structure from the top (i.e., near outlet), bottom (i.e., near inlet), and middle layers of columns colonized by SW3+1 community as well as those by SW1-only and SW3-only columns. The relative spatial distribution patterns of SW1 and SW3 in the top, middle, and bottom layers of the columns were qualitatively similar (Fig. 6) even as the abundance ratio between SW3 and SW1 increased between columns. The upper layer of the column always had a higher SW3 to SW1 abundance ratio than the lower part, suggesting that relative abundance of SW3 gradually decreased from the top of each column to the bottom.

However, we observed that the presence of SW1 in sand columns changed the spatial distribution of SW3, and vice versa. We calculated a top-to-bottom ratio for SW3 and SW1 to represent the vertical distribution of each strain in the column (Fig. S6 and S7). As the relative abundance of the other strain decreased, the top/bottom ratios of strains in the columns generally became more similar to the ratios observed in the strain-only column (Fig. S6 and S7). The SW3 top/bottom ratio in column 6, which had the largest ratio of SW3/SW1 abundance (SW3/SW1 = 119.4), was not significantly different from that of SW3-only columns (Fig. S6). However, the SW1 top/bottom ratios of the two SW1-only columns had a substantial amount of variation between them. Because of this variation, the top/bottom ratio of SW1 was more similar to that of the SW1-only columns when more SW1 was on the column (e.g., columns 1 to 3), but none of the ratios were significantly different from the SW1-only columns, even though the average ratio dropped from 0.65 (*P* = 0.32) to 0.16 (*P* = 0.07; Fig. S7). Therefore, the results demonstrate that the presence of a population in biofilm microbial communities can change the spatial distribution of other populations in the filter, although the variation in the top/bottom ratio can occasionally be fairly large in single-strain columns.

## DISCUSSION

### Effect of biofilm development of a single strain in porous media on E. coli retention.

E. coli retention in columns colonized by the five individual strains shows high reproducibility within replicate columns modified by the same strain and significant differences across columns modified by different strains. The results demonstrate that biofilm formation by environmental isolates in porous media can contribute to significant differences in bacterial retention. Although it is unclear what strain-specific characteristics influence the E. coli retention characteristics of our strains, biofilm surface characteristics have been found to influence particulate retention substantially (23, 27, 33, 37, 38). For example, our previous study measured the adhesive force between the biofilms of strains SW1 to 4 and a model colloid (carboxyl-modified-latex bead) and found that the adhesive force between the biofilms and the model colloid were strain-specific and positively correlated with model colloid retention efficiency in sand columns colonized by these biofilms (33). The adhesive force was attributed to variations in the biofilm biopolymer length and density and the strength of biopolymer-model colloid bonding. Significant differences in E. coli retention characteristics between biofilms formed by closely related bacteria (e.g., under the same genus) confirm the expectation that biofilm surface characteristics are not likely to be strongly constrained even at the fine taxonomic level, making it challenging to extrapolate retention characteristics of environmental bacteria based on their phylogenetic relationship to cultured representatives with known retention characteristics (52). The surface characteristics of bacterial contaminants also influence their retention. Thus, we do not expect that specific retention characteristics of these biofilm strains will hold broadly across various bacterial contaminants. The results suggest that environmental bacterial strains will have a wide range of retention characteristics against bacterial contaminants.

Although the model colloid, carboxyl-modified-latex bead, is often used as a surrogate for pathogenic microorganisms (e.g., oocysts, bacterial pathogen) because of their similarity in surface charge (53–57), the relative retention efficiency of E. coli was not the same as that of the model colloid (33) across these strains (Fig. S2). For example, PAO1 had been found with high retention efficiency of model colloid likely affected by straining (27, 33). Yet, in removing E. coli, straining was not observed, which could be related to less biofilm formation in columns and a smaller dimension of E. coli compared to that of the model colloid. SW4 had the highest retention efficiency of the model colloid across stormwater strains but a low E. coli retention efficiency that was not significantly different from that of SW2 but significantly lower than that of SW3. The variability might be attributed to differences in chemistry of the filtrate between the two studies, because yeast extract, which contains natural organic matter that influences colloid transport (20, 23), was not included in the stormwater media during E. coli retention measurement. The removal of yeast extract also decreased the ionic strength from 20 mM to 10 mM. Further, E. coli cells have surface characteristics different from those of the model colloid, influencing the interactions with the biofilm surface. Specifically, E. coli K-12 cells have biopolymers extending from the cell surface (40), while the model colloid has a bare surface likely free of polymers (33–35). Despite the difference in filtrate chemistry and particulate surface characteristics, SW1 and SW3 had similar efficiencies in removing the model colloid and E. coli, in which the difference between the retention of E. coli and the model colloid for the two strains was no greater than 4% (*P* > 0.05; Fig. S2). This suggests that biofilms formed by some strains might have similar retention characteristics for a range of particulate contaminants and could be relatively insensitive to the specific surface properties under similar filtrate chemistry. The presence of strains with high retention efficiencies in filters could potentially enhance the retention of a range of particulate contaminants.

### Effect of biofilm microbial composition on E. coli retention in porous media.

Biofilms in the environment are typically a combination of many different strains, yet how populations within biofilm microbial communities combine to influence bacterial retention has not previously been tested rigorously. Biofilms of environmental bacterial isolates display substantial differences in E. coli retention, suggesting that the impact of environmental biofilms with complex communities on bacterial retention could differ based on composition. As previous studies suggested (33, 37), biofilms influence particulate retention by covering and modifying the surface of porous media. The modification of porous media by biofilms with a complex microbial community might be as simple as physical partitioning of surface area by each biofilm community member or involve complex and complicated inter- or intraspecies interactions. The effect of physical partitioning of surface area might be similar to that of mixing porous media with different surface characteristics, in which bacterial retention efficiency of mixtures changes monotonically with the increase of the mixing ratio of a constituent medium until reaching the retention efficiency of that medium. For example, increasing the concentration of biochar mixed in porous media monotonically increased the bacterial retention efficiency (58, 59). In the present study, we observed that the increase of the abundance ratio of SW3 and PAO1 generally enhanced the E. coli retention efficiency in SW3+1 and PAO1+SW1 columns until reaching the retention efficiency in PAO1-only or SW3-only columns. This result suggests that the increase in relative abundance of a member in biofilm community might increase the proportion of porous media surface being covered by that population, hence changing the bacterial retention characteristics of the filter.

Further, the results suggest that the range of variation of E. coli retention efficiency in the two-strain community colonized columns is bounded largely by the bacterial retention characteristics of the two constituent community members. The E. coli retention efficiencies for all but two columns colonized by two-strain communities were within the range of the E. coli retention efficiencies of the single-isolate columns for the two isolates in the biofilm communities (Fig. 5). The two communities with retention efficiencies outside the range were small and nonsignificant. Thus, we cannot rule out the possibility that interactions between biofilm community members could influence the bacterial retention characteristics, allowing the retention to be greater or smaller than that of the individual strains alone.

### Implications for the influence of microbial community composition on bacterial retention in biofilm-colonized filters.

Previous studies have shown that the structure and diversity of microbial communities colonizing porous media undergo dynamic changes both spatially and temporally (60, 61), which is influenced by numerous factors, including influent water microbial community (62), chemical conditions (60, 63), and porous medium characteristics (64). Thus, we sought to determine whether the relative abundance and characteristics of individual strains within a biofilm community could be used to predict bacterial retention efficiency of the colonized filter. However, environmental biofilms consist of numerous uncharacterized species, manifesting complex inter- and intraspecies interactions that are hard to control and manipulate, making it challenging to quantify the impact of biofilm microbial composition on bacterial retention. Therefore, we simplified the biofilm communities to consist of only two characterized strains with various relative abundances and tested their impact on bacterial retention under well-controlled conditions. Using the model system, two problems were identified that would make prediction of bacterial retention from known characteristics and relative abundance difficult. First, as expected, bacterial retention efficiencies were very different even between closely related strains, making community characterization with 16S rRNA gene sequencing ineffective at discriminating between closely related strains with distinct bacterial retention characteristics. Second, the relationship between relative abundance of populations in a biofilm microbial community and their influence on bacterial retention characteristics is not straightforward. Specifically, while it was clear that changes in the relative abundance of strains in the biofilm altered E. coli retention rate for both pairs of strains, changes in relative abundance did not result in similar changes to retention between the two pairs of strains (i.e., different-shaped curves in Fig. 5). Therefore, our results demonstrate that individual strain characteristics within biofilm communities influence retention in a manner that is not dependent solely on their relative abundance. These insights demonstrate that it will be difficult to predict bacterial retention characteristics for more complex microbial communities, even under the best circumstances where individual retention characteristics of the strains are known.

Overall, the present study demonstrates that biofilm development by environmental isolates significantly altered E. coli retention efficiency, and variation in microbial composition in simplified biofilm communities influences the E. coli retention efficiency in porous media. Future studies should focus on testing how microbial composition of biofilms formed by complex mock communities and environmental bacteria influences the retention of various types of bacterial contaminants.

## MATERIALS AND METHODS

### Preparation of porous media.

The method of preparing porous media has been described in a previous study (33). Briefly, white quartz sand (Sigma-Aldrich, 50 to 70 mesh) was rinsed with deionized water to remove impurities, dried, and then autoclaved. Approximately 100 g of sand in total was added to each glass chromatography column (nominal diameter, 2.5 cm; length, 10 cm; bottom mesh size, 20 μm [Fisher Scientific]) with an identical dry-packing procedure. A total of 35 columns were prepared.

### Column pore volume measurement and sterilization.

After the columns were filled with sand, a peristaltic pump was connected to the bottom of the columns. The columns were saturated by forcing flow of 0.6% sodium hypochlorite solution (pH = 11.1) into the inlet. The direction of flow was upwards to reduce the creation of preferential flow paths in the columns. The pore volume (PV) of each column was determined by measuring the volume of sodium hypochlorite solution required to fill the column. The averages of pore volume, porosity, and specific surface area of all prepared columns under clean-bed conditions are 29 ± 2 ml, 0.44 ± 0.03, and 0.013 ± 0.001 μm^−1^ (see section 1 in the supplemental material for details about determining porosity and specific surface area). At least 1.5 PV of sodium hypochlorite solution was injected into each column. The remaining liquid was left in column overnight for sterilization. Then, the columns were rinsed to remove the hypochlorite solution by injecting approximately 1.5 PV sterile deionized water. The columns were ready for bacterial inoculation thereafter.

### Media for E. coli retention efficiency measurement and biofilm development.

A previously formulated synthetic stormwater (65) was used to suspend E. coli for E. coli retention efficiency measurement to simulate the general chemical characteristics of stormwater runoff (see section 2 in the supplemental material for details regarding E. coli suspension preparation). The synthetic stormwater consisted of 5 mM NaCl, 1 mM NaHCO_3_, 0.75 mM CaCl_2_, 0.30 mM Na_2_SO_4_, 0.15 mM NaNO_3_, 0.075 mM MgCl_2_, 0.07 mM NH_4_Cl, 0.02 mM Na_2_HPO_4_, 3 mg/liter hexane, 0.0015% (by weight) peptone, and 0.0011% meat extract, as well as 0.0003% urea (pH 6.9, ionic strength of 10 mM). In order to provide nutrients to speed biofilm development while still simulating the general characteristics of stormwater runoff, this recipe was amended with 3 g/liter yeast extract creating a nutrient-enriched synthetic stormwater (NESS) used in a previous experiment (33).

### Bacterial isolates for biofilm development and E. coli.

E. coli K-12 MG1655 (66) containing green fluorescent protein (67) (GenBank accession number MW349588) was used as a fecal indicator bacteria surrogate. Four stormwater bacterial strains and a laboratory bacterial strain, Pseudomonas aeruginosa PAO1 (abbreviated as PAO1, GenBank accession number: MW349586), were used to colonize sand columns used in E. coli retention measurements. The four stormwater bacterial strains include one strain from *Sphingobacterium* (SW1, GenBank accession number: MW349587), two strains from *Chryseobacterium* (SW2 and SW3, GenBank accession numbers: MW349584 and MW349583, respectively), and one strain from Pseudomonas (SW4, GenBank accession number: MW349585). These strains were randomly isolated from a set of laboratory-scale sand columns inoculated with stormwater runoff collected near Johns Hopkins Homewood campus (Baltimore, MD) in a previous study (68). Our earlier study demonstrated that under the growth condition identical to that of the present study (i.e., growth media chemistry, temperature and length of growth period), all strains can attach to glass coverslips and affect repulsion and adhesion characteristics to a model colloid (carboxyl-modified-latex beads) even in areas of the slide where cells are not present. The microscopy images of biofilms and detailed information about these strains (e.g., cell surface charge, cell surface hydrophobicity, biofilm surface biopolymer length) have been reported in the earlier study (33).

### Bacterial colonization of sand columns.

The first batch of experiments were designed to investigate how biofilms of single strains altered E. coli retention. A total of 10 columns were inoculated with one of four stormwater strains (SW1 to 4) or PAO1. Liquid culture of each strain was developed overnight in NESS at 37°C, reaching the cell density between 2 × 10^5^ to 5 × 10^7^ CFU/ml (see section 3 in the supplemental material for cell density estimation procedure and cell density of each inoculum). The overnight culture of each strain was used to inoculate two columns by injecting approximately 1.5 PV of the culture at a flowrate of approximately 0.69 ml/min (hydraulic retention time [HRT] of 42 min). The culture remained on the columns for 24 h under room temperature. To supply nutrient for biofilm growth, 1.5 PV of sterile NESS was injected into each column at 24 and 48 h at the same flowrate to inoculation after filtrating through a 0.2-μm filter connected near the inlet of each column to prevent biological contamination. NESS liquid remained on the columns under room temperature.

The second and third batches of experiments aimed to investigate how biofilms with various compositions of simplified communities alter E. coli retention. To colonize columns with simplified communities with varied compositions (i.e., the difference in the proportion of each population), we created column inocula by mixing two overnight cultures, either mixing SW3 with SW1 or mixing PAO1 with SW1 at mixing ratios ranging from approximately 10^2^:1 to 1:10^6^ (Table S3 and S4). The column inocula were added into columns to create differences in relative abundance over the experimental period. An antagonistic activity test (see details in section 4 of the supplemental material) was conducted beforehand to preclude the antagonistic relationship between SW1, SW3, PAO1, and E. coli in a pairwise manner. The second batch of experiments had seven columns inoculated with the mixtures of SW3 and SW1, along with two columns inoculated with only SW3 culture and another two with only SW1 culture. Similarly, the third batch of experiments had seven columns inoculated with the mixtures of PAO1 and SW1, along with two columns inoculated with only PAO1 culture and another two with only SW1 culture. For inoculation and nutrient supplement, we repeated the same procedure as described above. One column inoculated with SW3 and SW1 mixture and another inoculated with PAO1 and SW1 mixture were removed from analysis as discussed in the following section. In each batch of experiments, a clean-bed (no biofilm) column was included, in which only NESS with 50 mg/liter kanamycin sulfate antibiotic was added into columns during inoculation and nutrient supplying processes to maintain sterile conditions.

### E. coli retention measurements.

E. coli retention efficiency was measured for each column to evaluate retention behavior of E. coli in sand columns modified by different biofilms and clean-bed control. E. coli cells were suspended in synthetic stormwater at a cell density of approximately 10^4^ cells/ml (quantified by a flow cytometry; see details below). Before adding E. coli suspension into the columns, we injected 1.5 PV of synthetic stormwater to replace the remaining liquid in each column. The flow rate was stabilized to 0.69 ± 0.06 ml/min (HRT of 42 ± 4 min) during the injection. After this, the influent feed was immediately switched to E. coli suspension. Approximately 4 PV of E. coli-synthetic stormwater suspension was injected into each column. Effluent samples were taken sequentially at approximately every 0.5 PV. Influent samples were also taken during the test. The influent and effluent samples were preserved in a mixture containing 1% formalin and 25% glycerol at 4°C after collection and prior to analysis of E. coli cell density with a BD FACSCanto flow cytometer equipped with BD FACSDiva software (v8.0) at the Johns Hopkins University Homewood Flow Cytometry Resource Center. An internal counting standard, CountBright absolute counting beads (Molecular Probes), was mixed with each sample at a known ratio of 1:6. The mixture was then loaded onto the flow cytometer to count E. coli cells and internal counting beads passing through the flow cell. Green-fluorescent E. coli cells and brightly fluorescent CountBright beads (UV to 635 nm excitation and 385 to 800 nm emission) were distinguished from background noise using green fluorescence (bandpass filter 530/30) and side scatter (SSC). E. coli cell density in the sample was determined by the following equation:

$$\mathrm{cell density in sample}\;=\;\frac{\frac{\mathrm{coun}t_{\mathrm{cell}}}{\mathrm{coun}t_{\mathrm{countbright}}}\times \mathrm{conc}._{\mathrm{countbright}}\times V_{\mathrm{countbright}}}{V_{\mathrm{sample}}}$$

Where count_cell_ is the count of E. coli cells output by flow cytometer, count_countbright_ is the count of CountBright beads output by flow cytometer, conc._countbright_ is the concentration of CountBright beads provided by manufacturer, *V*_countbright_ is the volume of CountBright beads added to the sample, and *V*_sample_ is the volume of sample that was mixed with CountBright beads. A breakthrough curve was created by plotting normalized E. coli effluent concentration against the pore volume of E. coli injection. The E. coli retention efficiency was determined by subtracting normalized E. coli effluent concentration from unity. The breakthrough curve of one column from each community group (SW3+1 and PAO1+SW1) was removed from further analysis due to erroneous normalized effluent concentrations (significantly exceeding 100%; Table S5) likely resulting from errors in cell fixation before flow cytometer measurement.

### Design of strain-specific primers for SW1, SW3, and PAO1 for use in quantitative PCR.

Primers were designed to amplify a unique region of the 16S rRNA gene of SW1, SW3, and PAO1 to quantify the abundance for each strain. A total of 2 μl of 1:50 dilution of overnight culture of each strain was added into a PCR using 27 F (5′-AGAGTTTGATCCTGGCTCAG-3′) and 1492R (5′-GGTTACCTTGTTACGACTT-3′) as forward and reverse primer. A 10-minute interval of 98°C was used to heat the samples to facilitate cell lysis. To cover the whole length of the amplicon sequences (approximately 1,400 bp), each amplified product was sequenced from both ends by using 27F and 1492R as sequencing primers separately with Sanger sequencing at the Genetic Research Core Facilities at Johns Hopkins University. To align the sequences of both ends, a closely related sequence from NCBI’s *Bacteria* (and *Archaea*) 16S rRNA gene database identified using BLAST (69) was used as a reference sequence and aligned using MAFFT (70, 71). The most variable 15-base-pair-long regions among the three aligned sequences were identified and used to construct strain-specific primers. Two regions in each sequence that were most variable between the three sequences and had similar annealing temperatures were selected as forward and reverse primer sequences. For the purpose of adjusting annealing temperatures between forward and reverse primers, a few base pairs around the selected regions might also be included in primer sequences. The detailed information about the designed primers is presented in Table S1. The specificity of designed primers (i.e., amplification occurs only for the targeted strain) was evaluated using quantitative PCR (see section 5 in the supplemental material for details). The results indicate that the designed primers have high specificity in amplifying target strains (Table S2).

### Quantifying the abundance of populations within two-strain community columns.

Samples were collected from the columns as described previously (33). Briefly, after the E. coli retention efficiency was measured for each column, the columns were inverted to remove the remaining liquid by pumping sterilized air into the column from inlet. After the remaining liquid was drained, the sand in each column was sampled by forcing a homemade soil core sampler (created from a pipet by removing the tip) into a column from top to bottom. The rest of the sand in each column was homogenized before sampling to measure moisture content. The core samplers from the columns in the second batch of experiments were cut into three sections of equal length near inlet (bottom), middle, and near outlet (top). The sand in each section was homogenized before samples were taken. Sand from all the other columns was completely homogenized (not divided into three sections) before samples were taken. Approximately 0.25 to 0.5 g of sand samples was processed with a power soil DNA extraction kit (Qiagen) according to the manufacturer’s protocol. DNA from the columns colonized by SW1, PAO1, SW3, and their communities were analyzed with quantitative PCR using strain-specific primers. All quantitative PCRs were carried out using the following conditions: 35 cycles of amplification with each cycle having 98°C for 10 s for denaturation, 30 s for annealing at primer-specific temperatures (Table S1), and 72°C for 45 s for elongation. The quantification cycle (*C_q_*) values were converted into the concentration of amplicons using a standard curve from a known quantity of purified templates of each strain (see section 6 in the supplemental material for details). Strain ratios for two-strain communities were calculated by dividing the concentration of amplicons from one strain by the other. Although the ratio of gene copies does not equal the ratio of cells in the sample because of differences in the number of rRNA operons per cell between strains, the relative changes in the ratio of one strain to another between different simplified communities (e.g., fold changes) will be the same at the cell or gene level. Biological contamination of each column (e.g., dispersal of ambient bacteria into columns) was determined by digesting the amplicon products, amplified using universal 16S rRNA primers, with restriction enzymes following a method described in a previous study (33).

### Statistical tests.

The E. coli retention efficiencies of columns modified by single strains were tested with one-way analysis of variance (ANOVA) (72) to analyze the effect of colonization of different strains on the E. coli retention efficiency in sand columns. Welch *t* test was used to compare the columns colonized by different strains and different biofilm communities. All statistical tests were performed with R (73).

### Data availability.

The 16S rRNA gene sequences of SW1 to 4, PAO1, and E. coli used in this study were deposited in NCBI GenBank (GenBank accession numbers MW349583 to MW349588). Other data are available through the JHU Dataverse at https://doi.org/10.7281/T1/ZPJZJC.
