# Supplementary material for: Influence of Simplified Microbial Community Biofilms on Bacterial Retention in Porous Media under Conditions of Stormwater Biofiltration
Source: Microbiol Spectr. 2021 Oct 27;9(2):e01105-21. doi: 10.1128/Spectrum.01105-21 (PMC8549730; doi:10.1128/Spectrum.01105-21)
Supplement: SUPPLEMENTAL FILE 1 — Supplemental material. Download Spectrum.01105-21-s0001.pdf, PDF file, 1.8 MB [file spectrum.01105-21-s0001.pdf]

Supporting Information for:

Influence of simplified microbial community biofilms on bacterial retention in porous media under conditions of stormwater biofiltration

Yue Zhang[a], Yan He[a]*, Eric G. Sakowski[a]†, Sarah P. Preheim[a]#

[a] Department of Environmental Health and Engineering, Johns Hopkins University, 3400 N. Charles St., Baltimore, MD 21218

#Address correspondence to Sarah P. Preheim, spreheim@jhu.edu

*Present address: Yan He, Department of Energy, Environmental and Chemical Engineering, Washington University in St. Louis, One Brookings Drive, St. Louis, MO 63130

†Present address: Eric G. Sakowski, Department of Science, Mount St. Mary's University, 16300 Old Emmitsburg Rd, Emmitsburg, MD 21727

Supporting Methodological Information

1. Determination of porosity and specific surface area of clean-bed sand columns

The column porosity was calculated by dividing column pore volume (PV) by the volume of the column before filling sands (65 mL). To estimate the specific surface area of each column, we approximate the sand grain to be spherical and adapt an equation from a previous study (1) to calculate specific surface area:

$$a = \frac{6(1-\phi)}{d_c} \qquad \text{eq. 1}$$

where a is specific surface area, ϕ is porosity, and d_c is the average diameter of sand grains. The value of d_c is 263 μm, which was determined in our previous study (2).

2. *E. coli* suspension preparation

We adapted a method from Mohanty et al. (3) to prepare *E. coli* suspension. In brief, a loop from *E. coli* culture (store in 25% glycerol stock at -80 °C) was streaked on a NESS-agar plate that contains 50 mg/L kanamycin sulfate antibiotic and incubated at 37 °C for 24 hours. A single colony from the plate was transferred into 50 mL NESS containing 50 mg/L kanamycin sulfate antibiotic and incubated at 37 °C overnight. The developed *E. coli* culture was centrifuged to separate cell pellets from liquid, and the cell pellets were rinsed twice with synthetic stormwater before resuspending in synthetic stormwater at the final cell density of approximately 10^4 cells/mL.

3. Construct a calibration curve to estimate cell density in column inocula

The viable cell density in the column inocula for developing single-isolate biofilms (batch 1 columns) was estimated using calibration curves between quantitative PCR C_q values and viable cell concentrations (CFU/mL). To construct a calibration curve for each strain, 10 mL of overnight culture was developed as described above using Lysogeny Broth (LB) as culturing media. The overnight culture was diluted from 10^2 to 10^7 times using LB by ten-fold serial-

dilutions. An aliquot of 100 μL of each diluted culture was added onto three LB agar plates and then spread using an inoculation loop. The three plates serve as technical replicates for each dilution. The plates were incubated under 37 °C until clear single colonies can be seen (18 to 24 hours). Cell counts on triplicated plates ranging from 30 to 300 CFUs were averaged and used for calculating viable cell density at other dilution rates. The proxy of cell density in each diluted culture was quantified using a 35-cycle quantitative PCR with U515F-E786R (4) as primers (a technical replicate was included for each sample). The C_q values were then averaged between replicates and plotted against the corresponding cell density (Figure S9). The column inocula were diluted with nucleotide free water at varied ratios to produce C_q values within the linear range of their corresponding calibration curves. The estimated cell concentration of SW1, SW2, SW3, SW4 and PAO1 inocula was 5×10^7, 2×10^7, 3×10^7, 3×10^6 and 2×10^5 CFU/mL.

4. Antagonistic activity test

The antagonistic activity between SW1, SW3, PAO1 and *E. coli* was examined in a pairwise manner. 20 μL of overnight liquid culture of each strain was added onto a nutrient enriched synthetic stormwater (NESS)-agar plate and spread with sterile glass beads to create a bacterial lawn. A droplet (approximately 5 to 10 μL) of the overnight liquid culture of the other strains was added on the top of these plates to form a single colony. The NESS-agar plates were incubated under 37 °C for 2 to 3 days until bacterial lawn and colonies were visible. A zone of clearing around the single colony of later-added strain indicates the antagonistic activity between the pair of tested strains. No antagonistic activity was identified.

5. Test the specificity of SW1, SW3 and PAO1 primers

To verify the specificity of each pair of strain-specific primers, we conducted quantitative PCR to amplify same amount of DNA using both designed strain-specific primers and a pair of

universal 16S rRNA gene primers (U515F and E786R) (4). All quantitative PCR reactions were carried out using the following conditions: 35 cycles of amplification with each cycle having 98 °C for 10 seconds for denaturation, 30 seconds annealing at primer-specific temperatures (Table S1), and 72 °C for 45 seconds for elongation. The designed primers show high specificity in amplifying the target region (Table S2), facilitating quantitative analysis of the biofilm community structure.

6. Construct a calibration curve to quantify the concentration of amplicon

The DNA samples extracted from pure cultures of SW1, SW3 and PAO1 was amplified with their strain-specific primers. The amplicon samples were then running on a 1.5% agarose gel, and the sequences at the length expected by the annealing position of each primer set was cut and purified with Zymoclean Gel DNA Recovery Kit (Zymo Research). The purified amplicon samples of each strain were diluted to a gradient of concentrations. All diluted amplicon samples were added into quantitative PCR to produce C_q values. One diluted amplicon sample of each strain was analyzed on a Fragment Analyzer CE (Advanced Analytical) using a High Sensitivity NGS Fragment Kit (Agilent) to measure the concentration of amplicon. The measured value was converted with dilution ratios and then plotted against the corresponding C_q values to produce a calibration curve. The following equation (5) was used to calculate the primer amplification efficiency:

$$E = 10^{-s} - 1 \qquad \text{eq. 2}$$

Where E is primer amplification efficiency, *s* is the slope of each calibration curve in Figure S8. The amplification efficiencies were presented in Table S1 (average was taken if a primer has more than one calibration curve).

7. Test the reproducibility of *E. coli* removal in clean-bed columns

Two columns were packed, measured for pore volume and sterilized following the same procedure as described in the main text. They were injected with nutrient-enriched synthetic stormwater (containing 50 mg/L kanamycin sulfate antibiotic) in the same manner as we did for clean-bed control columns described in the main text. The liquid remained were kept in columns for 1 day to let conditioning film form before testing for *E. coli* removal. The columns were injected with 2 pore volumes of *E. coli*-synthetic stormwater suspension followed by 2 pore volumes of synthetic stormwater (non-*E. coli*). Effluent samples were collected sequentially at approximately every 0.5 pore volume. Samples were stored and analyzed in the same way as the other columns described in the main text. Results of this test are shown in Fig. S1.

Supporting Tables

Table S1. Detailed information about the strain-specific primers

Target strain	Forward primer sequence	Reverse primer sequence	Expected length of amplified region (bps)	Annealing temperature (°C)	Amplification Efficiency
**SW1**	TTACCGGCATCGGGAA	GCTGTCTATCGCCAACAT	644	64	64%
**SW3**	AATGGCATCATTTTAA	AAAGCCTATTTCTAAG	822	49	74%
**PAO1**	ACGTCCGGAAACGGG	ACCGTACTCTAGCTCAGT	497	64	79%

Table S2. Evaluation of the specificity of strain-specific primers using quantitative PCR.

	**SW1 primer**		**SW3 primer**		**PAO1 primer**		**U515F-E786R**		**Viable cell density**
**DNA**	C_q mean[1]	C_q std	C_q mean	C_q std	C_q mean	C_q std	C_q mean	C_q std	**(CFU/mL)[3]**
**SW1**	18.04	0.11	NA	NA	34.21*	NA	20.35	0.09	3×10^{7}
**SW3**	35.68*	NA[2]	13.28	0.25	NA	NA	17.88	0.17	6×10^{8}
**PAO1**	35.7*	NA	NA	NA	14.81	0.26	18.80	0.05	1×10^{8}

[1] C_q mean and C_q std are the mean and standard deviation of three technical replicates of the cycle number where the curve crossed a threshold in quantitative PCR. This value is inversely proportional to the amount of template in the sample.
[2] NA indicates that all replicates were below detection limit in 35 cycles of amplification.
[3] Viable cell density was estimated using calibration curves in Figure S9.
* indicates that two of the three technical replicates were below detection limit.

Table S3. The mixing ratios between SW3 and SW1 cultures in column inocula for developing SW3+1 community biofilms with varying community structures

Columns	Volumetric mixing ratios (SW3/SW1)
**SW3+1 (1)**	1.0×10^{-4}
**SW3+1 (2)**	2.7×10^{-6}
**SW3+1 (3)**	1.0×10^{-2}
**SW3+1 (4)**	1.0
**SW3+1 (5)**	9.0
**SW3+1 (6)**	59

Table S4. The mixing ratios between PAO1 and SW1 cultures in column inocula for developing PAO1+SW1 community biofilms with varying community structures

Column	Volumetric mixing ratio (PAO1/SW1)
**PAO1+SW1 (1)**	1.0×10^{-6}
**PAO1+SW1 (2)**	1.0×10^{-5}
**PAO1+SW1 (3)**	1.0×10^{-4}
**PAO1+SW1 (4)**	1.0×10^{-2}
**PAO1+SW1 (5)**	1.0
**PAO1+SW1 (6)**	99

Table S5. The *E. coli* normalized concentration of two discarded columns.

**Column**	**Pore volume of *E. coli* injection**	**Normalized effluent concentration of *E. coli* (%)**
**A column colonized by SW3+1 community**	0.56	755.43%
	0.96	2222.99%
	1.30	3098.38%
	1.63	443.47%
	2.03	279.99%
	2.42	163.83%
	2.80	157.47%
	3.22	179.34%
	3.70	144.35%
**A column colonized by PAO1+SW1 community**	0.23	0.06%
	0.69	71.05%
	1.11	206.68%
	1.52	178.37%
	2.03	172.51%
	2.53	114.90%
	2.94	89.29%
	3.42	71.06%
	3.85	69.44%

Supporting Figures

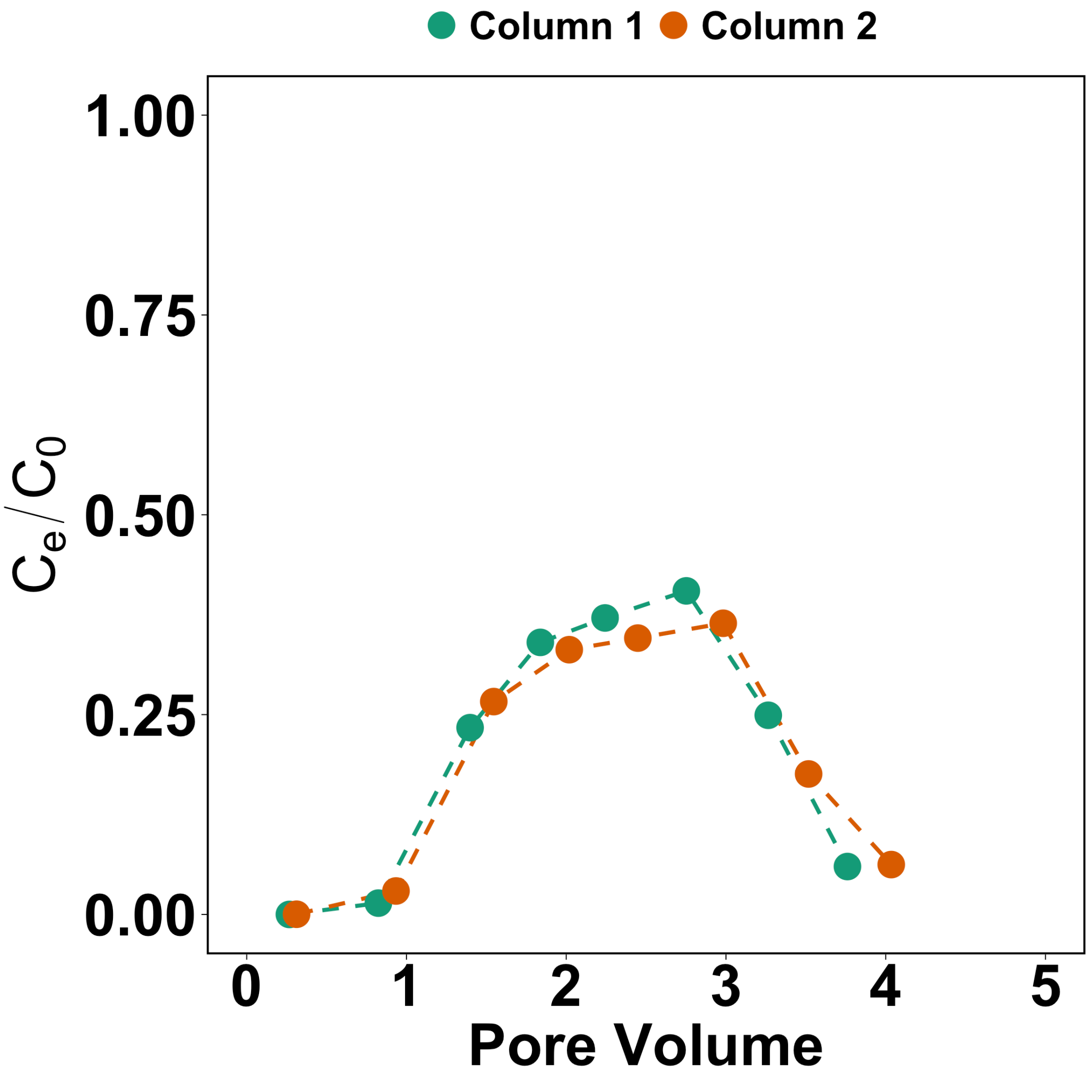

Figure S1. The breakthrough curves of *E. coli* in two clean-bed columns within the same batch. The columns were injected with 2 pore volumes of *E. coli* suspension followed by 2 pore volumes of synthetic stormwater.

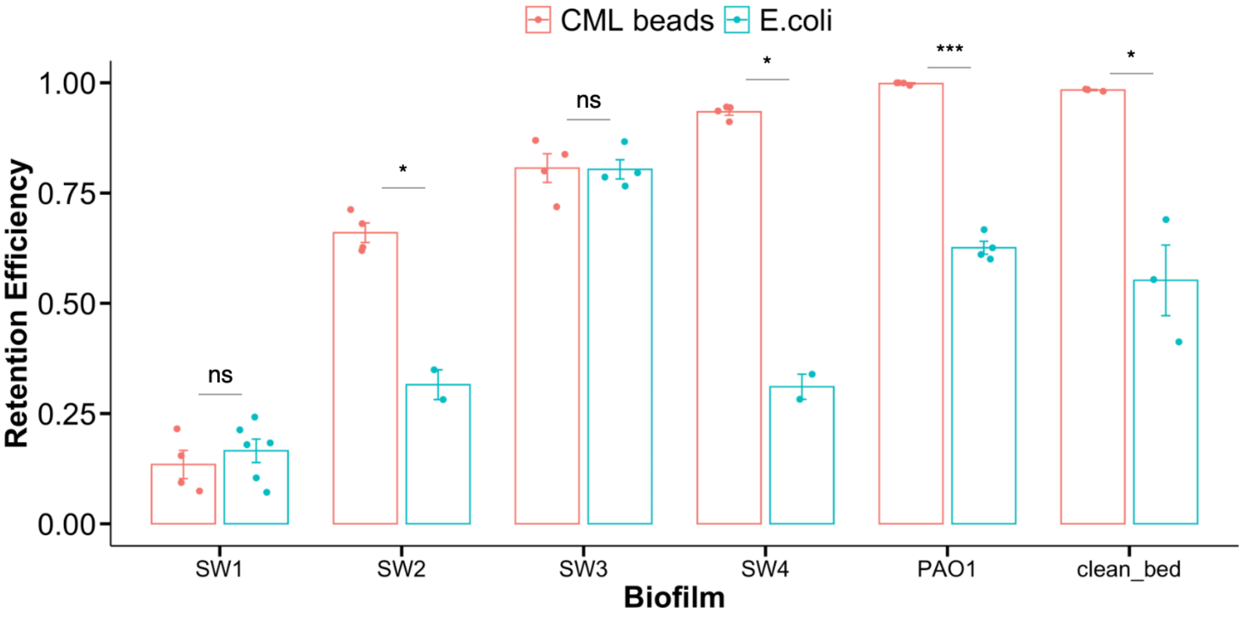

Figure S2. Retention efficiency of Carboxyl-modified-latex (CML) beads and *E.coli* of columns colonized by the five individual strains (SW1, SW2, SW3, SW4 and PAO1) and clean-bed columns. The data of CML retention efficiency was acquired from our earlier study (2), which was conducted using similar experimental settings except that the filtrate contained 3 g/L yeast extract. The error bars represent the standard deviation of replicate columns. The symbol above bars indicates significance of difference between bars in each pair (Welch t-test, ns = $p > 0.05$, * = $p < 0.05$, ** = $p < 0.01$, *** = $p < 0.001$).

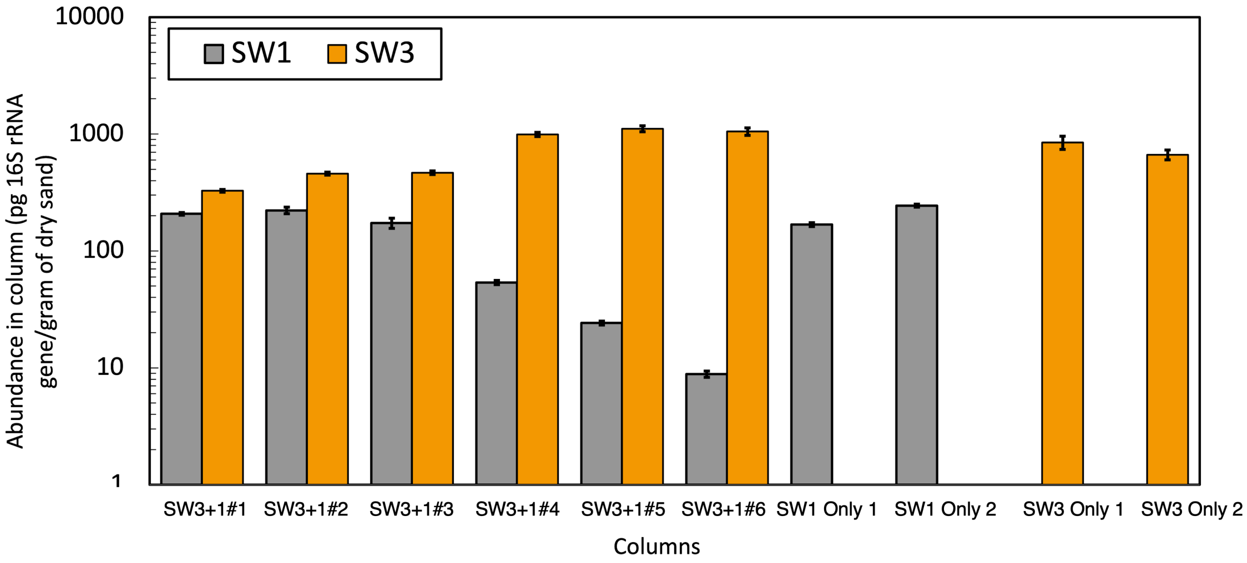

Figure S3. The concentration of 16S rRNA genes of SW1 and SW3 in SW3+1 community colonized columns and the SW1-only and SW3-only columns that included in the same batch. X-axis represent the 10 individual columns. Error bars represent the standard deviation of three technical replicate measurements in quantitative PCR.

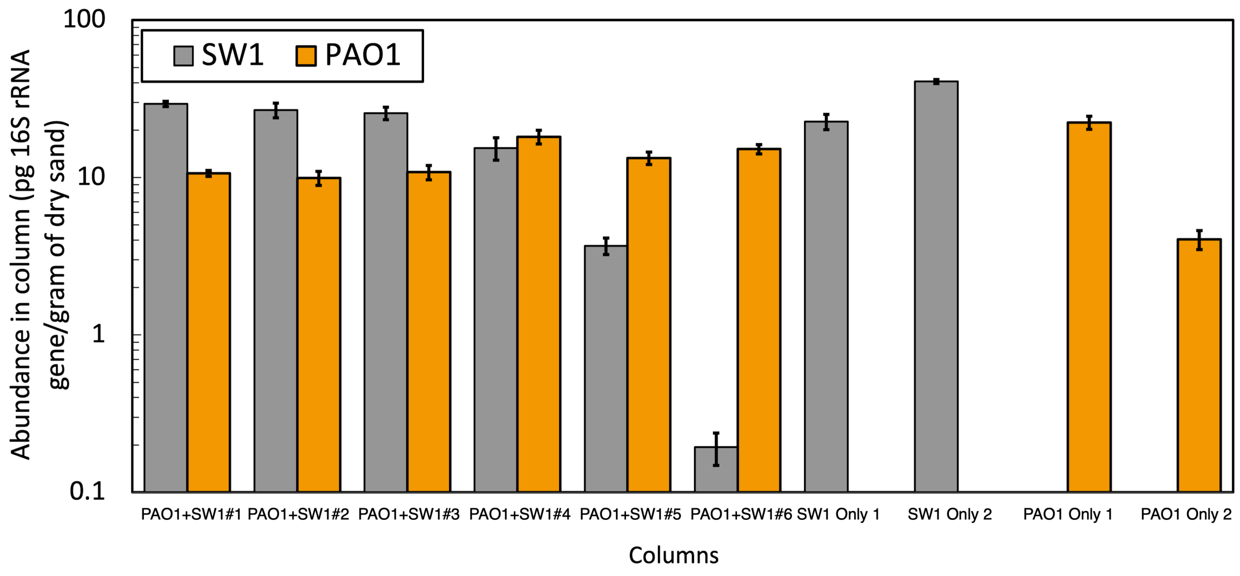

Figure S4. The concentration of 16S rRNA genes of SW1 and PAO1 in PAO1+SW1 community colonized columns and the SW1-only and PAO1-only columns that included in the same batch. X-axis represent the 10 individual columns. Error bars represent the standard deviation of three technical replicate measurements in quantitative PCR.

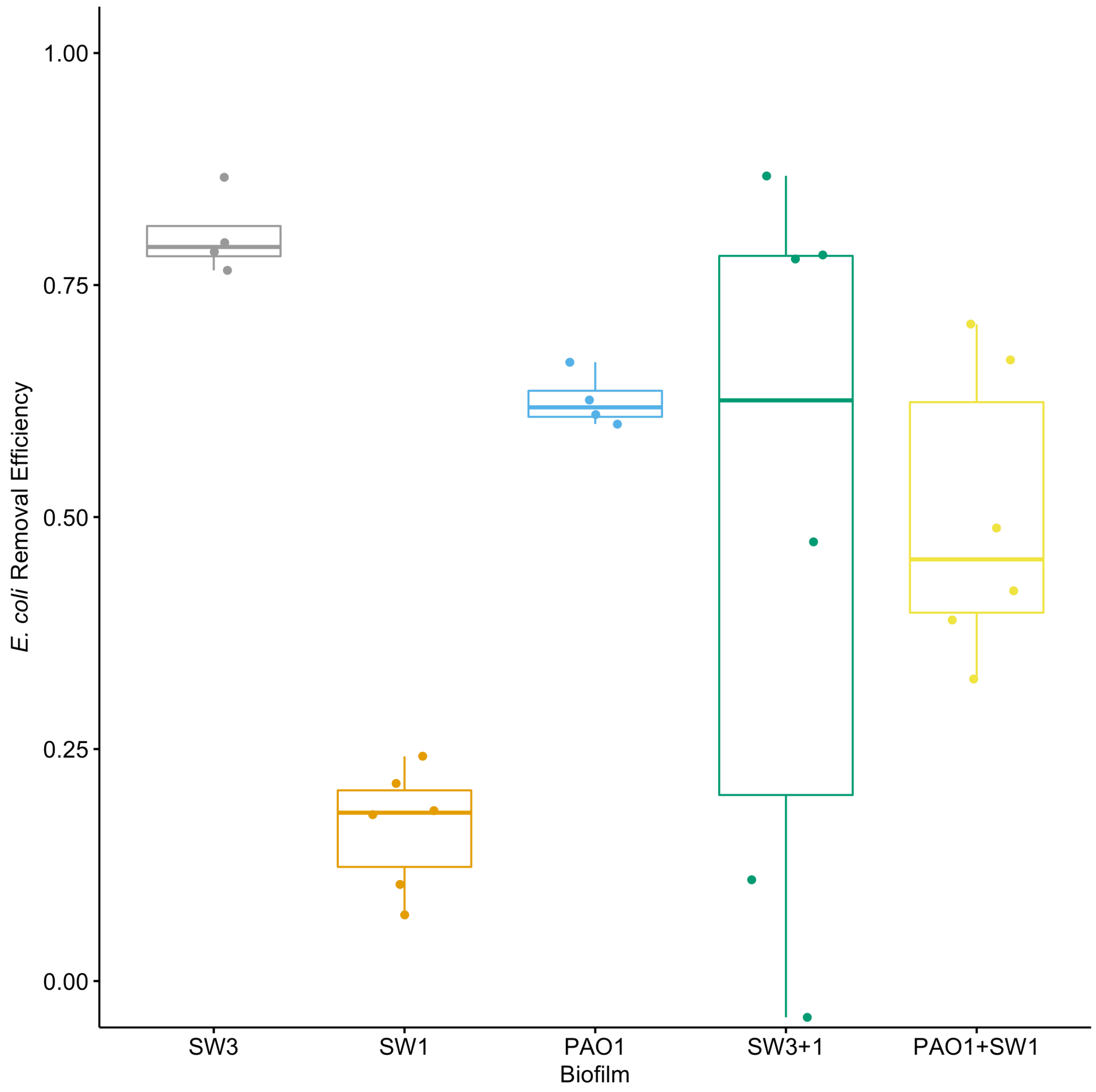

Figure S5. The comparison of *E. coli* removal efficiency of columns colonized by SW1, SW3, PAO1 and their two-strain communities. Dots represent individual measurements in different column experiments along with box and whisker plots of associated median, quartile and extreme values.

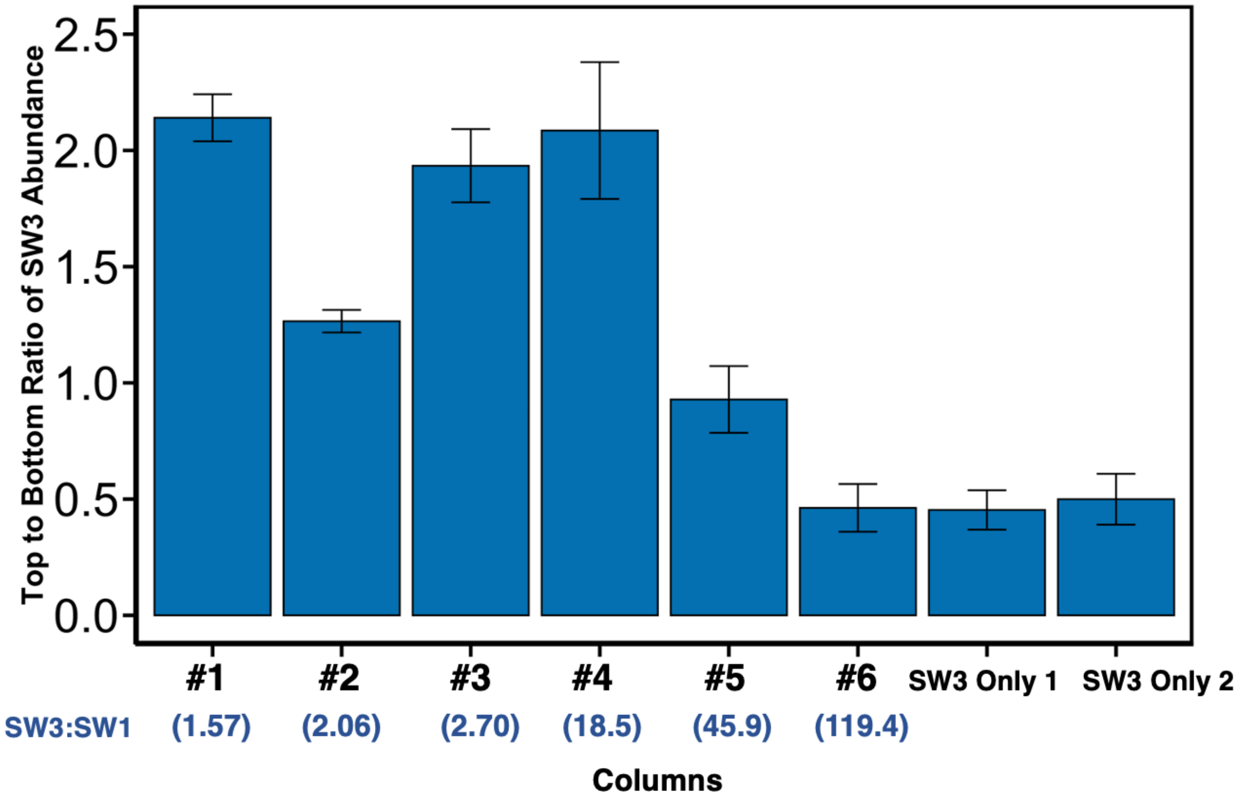

Figure S6. The top to bottom ratio of 16S rRNA gene concentration of SW3 in columns colonized by SW3+1 communities and by only SW3. Error bars represent the standard deviation of three technical replicate measurements in quantitative PCR. The numbers in the parentheses indicate the SW3:SW1 abundance ratio of the column.

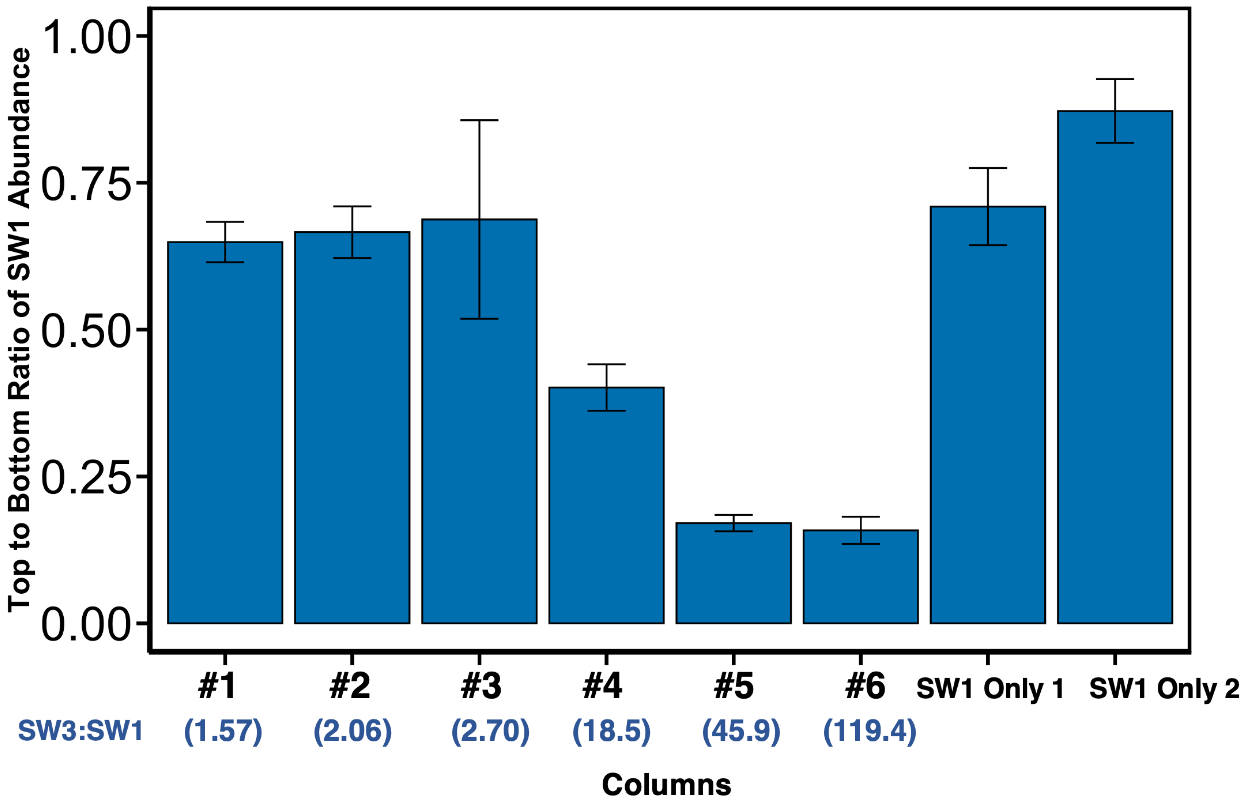

Figure S7. The top to bottom ratio of 16S rRNA gene concentration of SW1 in the columns colonized by SW3+1 communities and by only SW1. Error bars represent the standard deviation of three technical replicate measurements in quantitative PCR. The number in the parentheses indicate the SW3:SW1 abundance ratio of the column.

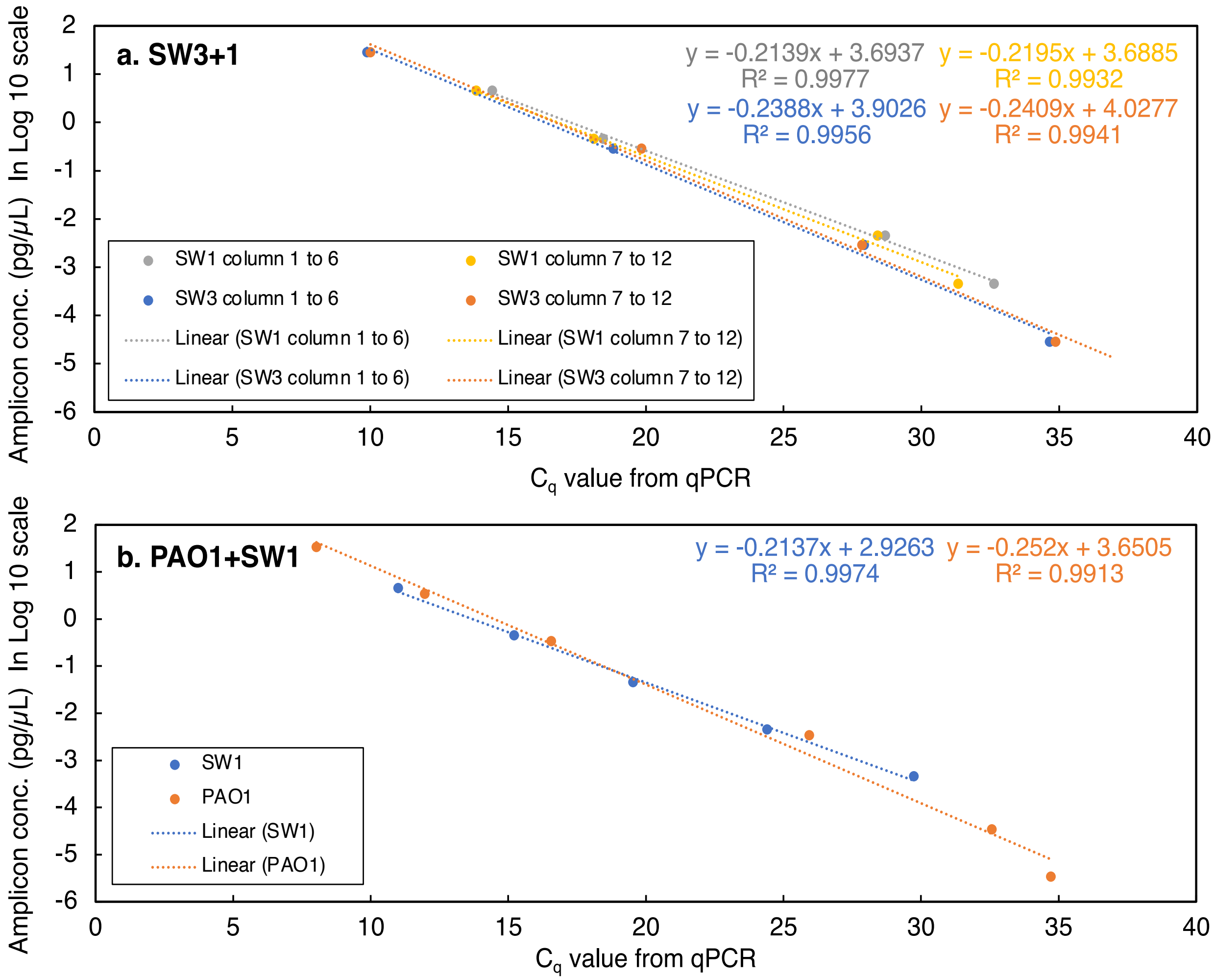

Figure S8. The calibration curve of amplicon concentrations for strain SW1-4 and PAO1. X-axis shows the C_q value obtained from a 35-cycle quantitative PCR reaction using strain-specific primers; y-axis shows the concentration of amplicons quantified using a fragment analyzer.

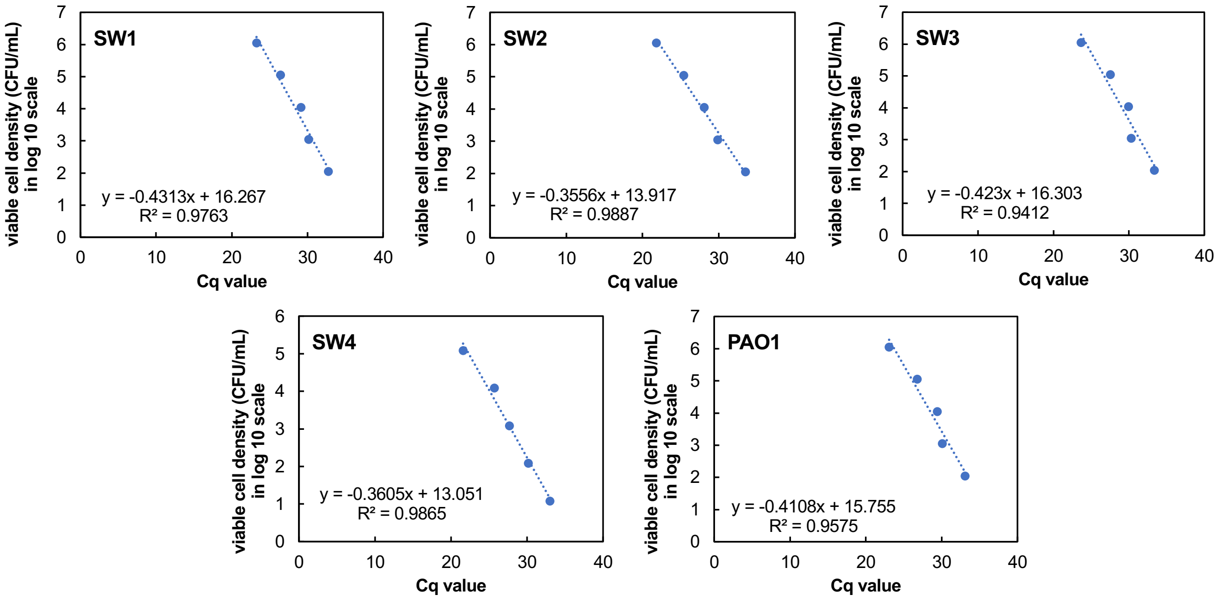

Figure S9. The calibration curve of viable cell count for SW1-4 and PAO1. X-axis shows the C_q values produced from a 35-cycle quantitative PCR reaction using U515F-E786R as primers; y-axis shows the viable cell density (CFU/mL) obtained by plate count.

Supporting References

1. Bozorg A, Gates ID, Sen A. 2015. Impact of biofilm on bacterial transport and deposition in porous media. Journal of Contaminant Hydrology 183:109-120.
2. Zhang Y, Wayner CC, Wu S, Liu X, Ball WP, Preheim SP. 2021. Effect of Strain-Specific Biofilm Properties on the Retention of Colloids in Saturated Porous Media under Conditions of Stormwater Biofiltration. Environmental Science & Technology doi:10.1021/acs.est.0c06177.
3. Mohanty SK, Torkelson AA, Dodd H, Nelson KL, Boehm AB. 2013. Engineering Solutions to Improve the Removal of Fecal Indicator Bacteria by Bioinfiltration Systems during Intermittent Flow of Stormwater. Environmental Science & Technology 47:10791-10798.
4. LANE DJ. 1991. 16S/23S rRNA sequencing. Nucleic acid techniques in bacterial systematics:115-175.
5. Kralik P, Ricchi M. 2017. A Basic Guide to Real Time PCR in Microbial Diagnostics: Definitions, Parameters, and Everything. Frontiers in Microbiology 8:108.
